# The Biogeographical Distribution of Benthic *Roseobacter* Group Members along a Pacific Transect Is Structured by Nutrient Availability within the Sediments and Primary Production in Different Oceanic Provinces

**DOI:** 10.3389/fmicb.2017.02550

**Published:** 2017-12-18

**Authors:** Marion Pohlner, Julius Degenhardt, Avril J. E. von Hoyningen-Huene, Bernd Wemheuer, Nora Erlmann, Bernhard Schnetger, Thomas H. Badewien, Bert Engelen

**Affiliations:** ^1^Paleomicrobiology Group, Institute for Chemistry and Biology of the Marine Environment, University of Oldenburg, Oldenburg, Germany; ^2^Genomic and Applied Microbiology and Göttingen Genomics Laboratory, Institute of Microbiology and Genetics, University of Göttingen, Göttingen, Germany; ^3^Microbiogeochemistry Group, Institute for Chemistry and Biology of the Marine Environment, University of Oldenburg, Oldenburg, Germany; ^4^Group “Marine Sensor Systems”, Institute for Chemistry and Biology of the Marine Environment, University of Oldenburg, Oldenburg, Germany

**Keywords:** diversity, next-generation sequencing, CARD-FISH, qPCR, RV Sonne

## Abstract

By now, only limited information on the *Roseobacter* group thriving at the seafloor is available. Hence, the current study was conducted to determine their abundance and diversity within Pacific sediments along the 180° meridian. We hypothesize a distinct biogeographical distribution of benthic members of the *Roseobacter* group linked to nutrient availability within the sediments and productivity of the water column. Lowest cell numbers were counted at the edge of the south Pacific gyre and within the north Pacific gyre followed by an increase to the north with maximum values in the highly productive Bering Sea. Specific quantification of the *Roseobacter* group revealed on average a relative abundance of 1.7 and 6.3% as determined by catalyzed reported deposition-fluorescence *in situ* hybridization (CARD-FISH) and quantitative PCR (qPCR), respectively. Corresponding Illumina tag sequencing of 16S rRNA genes and 16S rRNA transcripts showed different compositions containing on average 0.7 and 0.9% *Roseobacter*-affiliated OTUs of the DNA- and RNA-based communities. These OTUs were mainly assigned to uncultured members of the *Roseobacter* group. Among those with cultured representatives, *Sedimentitalea* and *Sulfitobacter* made up the largest proportions. The different oceanic provinces with low nutrient content such as both ocean gyres were characterized by specific communities of the *Roseobacter* group, distinct from those of the more productive Pacific subarctic region and the Bering Sea. However, linking the community structure to specific metabolic processes at the seafloor is hampered by the dominance of so-far uncultured members of the *Roseobacter* group, indicating a diversity that has yet to be explored.

## Introduction

The *Roseobacter* group within the family *Rhodobacteraceae* consists of nearly 90 genera and approximately 300 species (Pujalte et al., 2014; http://www.bacterio.net/). They thrive in a broad variety of marine habitats (Luo and Moran, 2014) and were found free-living in seawater (Giovannoni and Stingl, 2005), associated with micro and macro algae, marine sponges and invertebrates (González et al., 2000; Ivanova et al., 2004) as well as in biofilms, sea ice and sediments (Brinkmeyer et al., 2003; Inagaki et al., 2003). This wide habitat range is attributed to the broad metabolic versatility of different members within the group (Wagner-Döbler and Biebl, 2006). Most of the *Roseobacter*-affiliated bacteria are characterized as ecological generalists (Newton et al., 2010). However, some *Roseobacter* species are specialized to e.g., aerobic anoxygenic phototrophy, sulfur transformations, aromatic compound degradation or secondary metabolite production (Shiba, 1991; González et al., 1996, 1999; Brinkhoff et al., 2004). Due to their metabolic flexibility, the *Roseobacter* group can contribute in high proportions to the bacterial community composition in various marine habitats. For instance, they can account for up to 16% of bacterioplankton communities in polar and temperate waters (Selje et al., 2004). In a North Atlantic algal bloom, on average 23% of all 16S rRNA genes were affiliated to members of the *Roseobacter* group (González et al., 2000). Even subclasses within this group, e.g., the *Roseobacter* clade-affiliated cluster, can account for 36% of bacterial communities in coastal Antarctic regions (Giebel et al., 2009).

However, most studies on the abundance and diversity of the *Roseobacter* group were conducted on pelagic samples (Giebel et al., 2011; Wemheuer et al., 2014; Billerbeck et al., 2016; Zhang et al., 2016). In contrast, the distribution of this group in sediments is far less understood, even though 28% of all described *Roseobacter*-affiliated species which were known in 2014 are of benthic origin (Pujalte et al., 2014). This may be due to the fact that many studies in sediment microbiology focus on specific metabolic processes such as fermentation, nitrogen cycling, sulfate reduction, methanogenesis, or anaerobic methane oxidation (Orphan et al., 2001; Llobet-Brossa et al., 2002; Mills et al., 2008; Graue et al., 2012b). In diversity studies on marine sediments, the *Roseobacter* group is frequently neglected as their relative proportion on the benthic communities is often <10%. However, direct quantifications of the *Roseobacter* group at the sediment surface of North Sea tidal flats by CARD-FISH showed that their cell numbers exceed those in the pelagic environment by a factor of 1000 (Lenk et al., 2012). In reports on other coastal sediments, the *Roseobacter* group accounts for 1 to 4% of the entire bacterial community (Buchan et al., 2005; Kanukollu et al., 2016) or even 10% in brackish river sediments (González et al., 1999). Assuming that surface sediments contain approximately 10^9^ cells × cm^−3^, their absolute abundance is still orders of magnitudes higher than in corresponding water samples, which usually exhibit 10^6^ cells × ml^−1^ (Kallmeyer et al., 2012). The *Roseobacter* group does not only differ numerically between benthic and pelagic systems, but also in the community structure (Stevens et al., 2005). In a recent study on the distribution of the *Roseobacter* group in coastal North Sea sediments and water samples, we have shown that the diversity within the group increases from the sea surface to the seafloor revealing specific compositions of free-living and attached fractions (Kanukollu et al., 2016).

**Figure 1:**
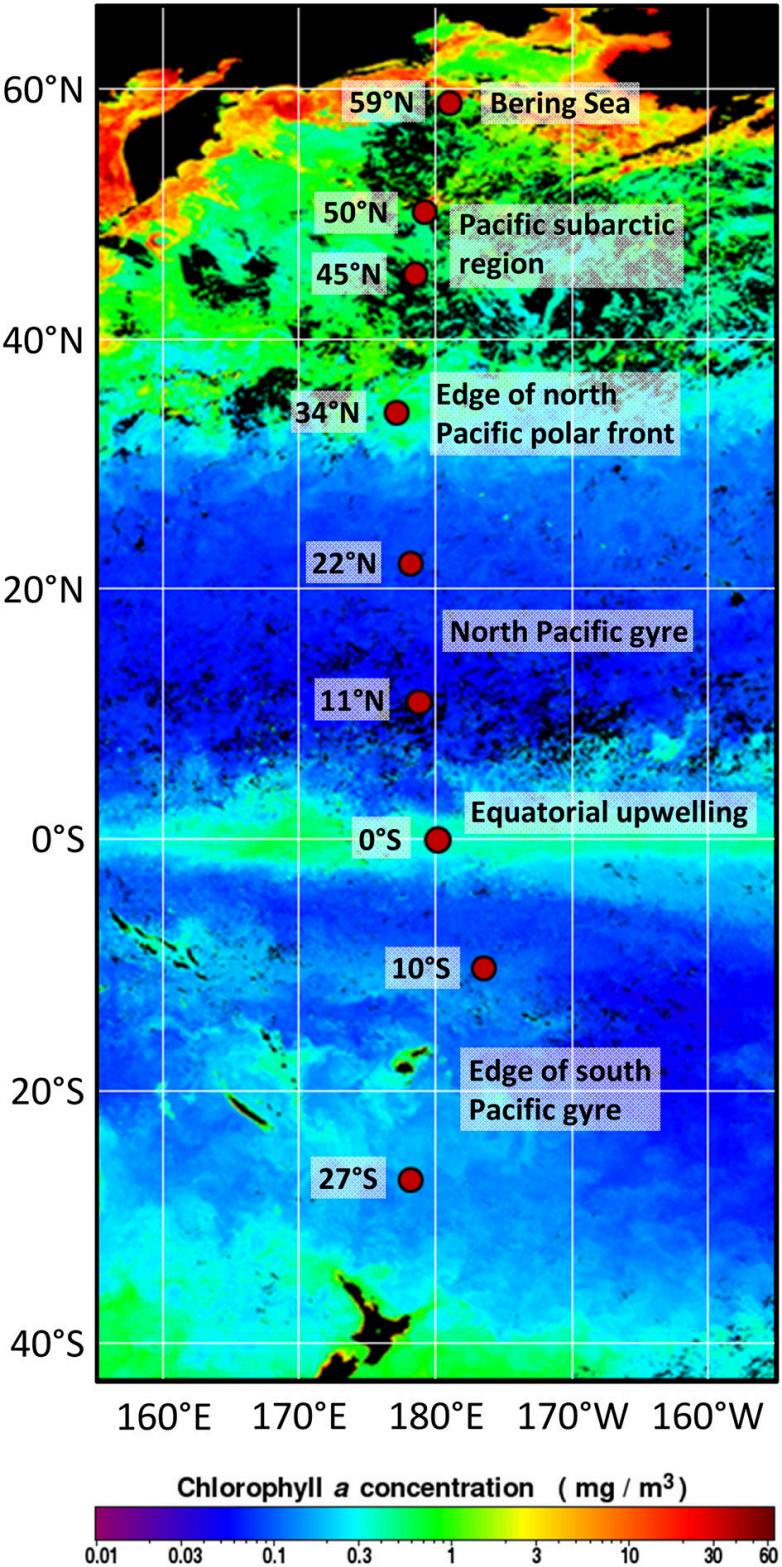
Positions of the investigated sampling sites in the Pacific along the 180° meridian from 27°S to 59°N. Sediment samples were taken at various water depths during RV Sonne expedition SO248, crossing the main oceanic provinces as classified by Longhurst (2007). The satellite map of chlorophyll *a* concentrations at the sea surface during May 2016 was modified from OceanColorWeb (Gene Carl Feldman; https://oceancolor.gsfc.nasa.gov/cgi/l3).

As all other benthic studies on the *Roseobacter* group were performed on relatively eutrophic coastal sites or high-energy systems such as hydrothermal vents (Buchan et al., 2005), we now describe their abundance, distribution and diversity in oligotrophic deep-sea sediments. In this study, we investigated sampling sites of a Pacific transect (Figure 1) spanning six distinct oceanic provinces as defined by Longhurst (2007). These provinces are principally formed by ocean circulation patterns leading to varying nutrient concentrations in the water column. The availability of nutrients directly effects the phytoplankton composition and pelagic bacterial communities (Longhurst, 2007). For instance, the north and south Pacific gyres are established by circular currents that cut off these oceanic regions from a continental nutrient inflow. These oligotrophic conditions lead to low primary production, extremely low sedimentation rates of organic matter and therefore to decreasing cell numbers and associated microbial activities at the seafloor (Rullkötter, 2006; Kallmeyer et al., 2012; Røy et al., 2012). Between the mid-ocean gyres, the upwelling along the equator transports nutrients to the sea surface and thereby stimulates primary production in the photic zone (Schulz and Zabel, 2006). Further north, within the Pacific subarctic region, water column productivity increases toward the Bering Sea. In the highly productive Bering Sea, proximity to land in combination with the coastal runoff leads to increased phytoplankton activities (Longhurst, 2007). The stimulation of primary production causes high organic matter sedimentation and therefore elevated nutrient concentrations at the seafloor (Schulz and Zabel, 2006; Wehrmann et al., 2011).

Our transpacific survey now offers the opportunity to investigate the *Roseobacter* group in deep-sea sediments exhibiting a wide range of environmental conditions from low to relatively high nutrient concentrations. We hypothesize a distinct biogeographical distribution of benthic *Roseobacter* group members corresponding to the composition and nutrient availability within the sediments and primary production in the water column. Thus, sediments were sampled during RV Sonne expedition SO248 along the 180° meridian covering the main oceanic provinces of the central Pacific. In total, seafloor samples (0–1 cm below seafloor, cmbsf) were collected at nine sites from 27°S to 59°N exhibiting water depths of 3,258–5,909 m. Total cell counts were compared to the specific abundance of *Bacteria* and the *Roseobacter* group as quantified by CARD-FISH and qPCR. Next-generation sequencing of 16S rRNA genes and 16S rRNA transcripts revealed deeper insights into the diversity and distribution of the *Roseobacter* group within Pacific sediments. Finally, sediments were characterized by their geochemical composition to correlate the distribution patterns of *Roseobacter* group members to the different environmental settings.

## Materials and methods

### Origin and sampling of sediments

Sediment samples were collected in May 2016 during RV Sonne expedition SO248 along a transect from Auckland, New Zealand to Dutch Harbor, Alaska, USA (Figure 1). The transect included very productive as well as highly oligotrophic regions in the Pacific Ocean and covered the main oceanic provinces along the 180° meridian from 27°S to 59°N (Table 1). Sediment cores were taken by a multicorer (Octopus, Germany) at nine sites, exhibiting water depths between 3,258 and 5,909 meter below sea level (mbsl). Subsampling of the sediment surface (0–1 cmbsf) was performed using sterile, cutoff syringes, and rhizons for porewater collection (Rhizosphere, Netherlands; Seeberg-Elverfeldt et al., 2005). Porewater was stored at −20°C, while samples for DNA and RNA extraction were frozen at −80°C for molecular analysis. Samples for cell counting (0.5 cm^3^) and CARD-FISH (1 cm^3^) were fixed for at least 1 h with 3% glutaraldehyde or 3% formaldehyde following the protocols of Lunau et al. (2005) and Ravenschlag et al. (2001), respectively. Afterwards, samples were centrifuged at 13,000 rpm for 1 min and washed two times with 1x TAE-buffer (40 mM NaCl, 1 mM Na_2_EDTA, pH 7.4 adjusted with acetic acid). Finally, samples were stored in TAE:EtOH (1:1) at −20°C.

**Table 1:** Origin of sediment samples.

**Site**	**Oceanic province**	**Latitude**	**Longitude**	**Depth (mbsl)**	**Sum chlorophyll (mg/m^3^)**
27°S	Edge of south Pacific gyre	26° 60′ S	178° 14′ E	4185	42
10°S	Edge of south Pacific gyre	10° 20′ S	176° 29′ W	4130	88
0°S	Equatorial upwelling	0° 2′ S	179° 59′ W	5285	102
11°N	North Pacific gyre	10° 58′ N	179° 0′ E	5402	67
22°N	North Pacific gyre	21° 58′ N	178° 19′ E	3258	49
34°N	Edge of north Pacific polar front	33° 58′ N	177° 21′ E	3486	52
45°N	Pacific subarctic region	45° 0′ N	178° 45′ E	5909	123
50°N	Pacific subarctic region	49° 60′ N	179° 33′ E	5621	121
59°N	Bering Sea	58° 55′ N	178° 56′ W	3300	119

*The oceanic provinces and water depths are indicated. Chlorophyll data were summed up for the top 500 m of the water column*.

### Measurement of chlorophyll concentrations

During the expedition, chlorophyll concentrations were measured using a CTD-rosette equipped with a fluorometer (FluoroWetlabECO_AFL_FL, SN: FLNTURTD-4111). Data were recorded and stored using the standard software Seasave V 7.23.2 and processed by means of ManageCTD. The sum of chlorophyll measured in the top 500 m of the water column was used to characterize the productivity in the water column at the different sampling sites along the transect. Raw data are available at PANGAEA: https://doi.pangaea.de/10.1594/PANGAEA.864673 (Badewien et al., 2016).

### Geochemical analyses of bulk sediments and porewaters

Sediments were freeze-dried (Beta 1-8 LDplus, Christ, Germany) and ground as well as homogenized in a Mixer Mill MM 400 (3 Hz, 50 min; Retsch, Germany). Total carbon (TC) and sulfur (S) was analyzed with an elemental analyzer (Eltra CS-800, Germany) with a precision and accuracy of <3% (1σ). Inorganic carbon (IC) was analyzed using an acidification module (Eltra CS-580) and the content of total organic carbon (TOC) was calculated by difference (TC–IC). Major and trace element analysis in sediments were performed by wavelength dispersive X-ray fluorescence (XRF, Panalytical AXIOS plus) on fused borate glass beads (detailed method in data repository of Eckert et al., 2013). For XRF measurements, accuracy and precision were tested with several SRM's being <4 rel-% (1σ). Nutrient concentrations of ammonium (NH_4_), nitrate (NO_3_), phosphate (PO_4_) and silicic acid in porewaters were determined photometrically using the Multiscan GO Microplate Spectrophotometer (Thermo Fisher Scientific, USA). The method for measuring ammonium was modified after Benesch and Mangelsdorf (1972), NO_3_ was quantified with the method described by Schnetger and Lehners (2014). Concentrations of PO_4_ and silicic acid were determined following the protocol of Grasshoff et al. (1999). Precision and accuracy were tested using solutions of known analyte concentrations (independently prepared) and were <10% (1σ).

### Total cell counts

To compare total cell counts to previous studies on the abundance of benthic prokaryotes (Kallmeyer et al., 2012; Engelhardt et al., 2014), counting was conducted by epifluorescence microscopy using SYBR Green I as fluorescent dye. The protocol was performed according to Graue et al. (2012a) with slight modifications. Those included sonication (three times for 1 min followed by 1 min cooling on ice to prevent overheating), settling of the sample for 1 min to remove sand grains and an initial 10x to 50x dilution of the supernatant with TAE:EtOH (1:1). 10 μl of the diluted sample were dispensed on a microscopic slide and dried. A SybrGreen I staining solution was prepared using 5 μl of the concentrated SybrGreen I stock solution (Molecular Probes, Eugene, USA) diluted in 200 μl moviol mounting medium (Lunau et al., 2005). A freshly prepared 1 M ascorbic acid solution, dissolved in TAE buffer was added at a final concentration of 1% as an antioxidant. Cells were stained with 8 μl of the SybrGreen I staining solution. Counting was performed using an epifluorescence microscope (Zeiss, Germany) and twenty randomly selected fields were counted for each sediment sample.

### Catalyzed reported deposition-fluorescence *in situ* hybridization (CARD-FISH)

CARD-FISH quantification was performed as described by Pernthaler et al. (2002). First, 200 μl sediment slurry was diluted 1:4 with 1x PBS (phosphate-buffered saline, 137 mM NaCl, 2.7 mM KCl, 19 mM NaH_2_PO_4_, 1.8 mM KH_2_PO_4_, pH 7.4) and sonicated for 15 min at 35°C. The sample was further diluted 1:200 in 1x PBS, filtered through a 0.2 μm filter (Nucleopore, Whatman) and washed with 30 ml of sterile 1x PBS. Hybridization was carried out with 35% formamide at 46°C. The horseradish peroxidase-labeled *Roseobacter*-specific probe Roseo536 (5′-CAA CGC TAA CCC CCT CCG-3′ plus competitor; Brinkmeyer et al., 2000) was diluted 1:100 in hybridization buffer. Samples were counterstained using 3 μl of DAPI (4′,6-diamidino-2-phenylindole) and stored at −20°C until microscopic analysis. Microscopic images were taken semi-automatically at 55 randomly chosen spots using an epifluorescence microscope (AxioImager.Z2m, software package AxioVisionVs 40 V4.8.2.0; Carl Zeiss, Germany). Signals were counted using the automated image analysis software ACMEtool3 (M. Zeder; www.technobiology.ch).

### Extraction of nucleic acids

DNA extraction from sediment samples was performed using the DNeasy PowerSoil Kit (Qiagen, Germany) according to manufacturer's instructions. DNA was extracted from 0.5 cm^3^ sediment and eluted from the columns using 30 μl of PCR-grade water. RNA was extracted from 1 cm^3^ sediment using the AllPrep DNA/RNA Mini Kit (Qiagen, Germany) following the manufacturer's protocol with some modifications prior to the disruption and homogenization of the cells: an equal volume (1 ml) of Bacterial RNA protect (Qiagen, Germany) was mixed with the sediment by vortexing for 5 s to prevent RNase-digestion. After incubation for 5 min and centrifugation (15 min, 10,000 rpm), the supernatant was discarded. Then, 1 g of zirconia-silica beads (diameter: 0.1 mm; Roth, Germany) and 700 μl of RLT-buffer (amended with 0.7 μl β-mercaptoethanol) were added to the pellet. The cells were mechanically disrupted by vigorously shaking using a Mini-Beadbeater (BiospecProducts, USA) for 90 s. The homogenized mixture was centrifuged for 3 min at 13,400 rpm and the supernatant was transferred to an AllPrep DNA spin column provided with the kit. After centrifugation for 30 s at 13,400 rpm, the flow through was mixed with 470 μl of ice cold absolute ethanol and used for RNA purification on the RNeasy Mini spin column following the original protocol. Finally, the RNA was eluted in 40 μl of PCR-grade water. The concentration and purity of the DNA and RNA extracts was determined spectrophotometrically (Nanodrop 2000c, Thermo Fisher Scientific, USA).

### Quantification of 16S rRNA gene targets

To determine the amount of 16S rRNA gene targets in the sediment samples, quantitative PCR (qPCR) was performed using the DyNAmo HS SYBR Green qPCR Kit (Thermo Fisher Scientific, USA) and the Light Cycler480II (Roche, Germany). The reaction was performed in 25 μl setups: 12.5 μl 2x master mix (supplied by the kit), 0.5 μl BSA (10 mg/μl), 0.5 μl of forward and 0.5 μl of reverse primer (each 10 pmol/μl), 1 μl PCR-grade water and 10 μl of template DNA (1:100 and 1:200 dilutions for EUB, 1:100 dilution for Roseo). For quantification of *Bacteria* the specific primer pair 519f/907r (Lane, 1991; Muyzer et al., 1995) was used. The *Roseobacter* group was quantified by the *Roseobacter*-specific primers Roseo536f (reverse complementary to Roseo536r; Brinkmeyer et al., 2000) and GRb735R (Giuliano et al., 1999). Cycler settings for the *Bacteria*-specific qPCR included an activation step for 15 min at 95°C, followed by 50 cycles of denaturation for 10 s at 94°C, annealing for 20 s at 55°C and elongation for 30 s at 72°C. Afterwards a constant temperature was set for 2 min at 50°C with a subsequent melting curve analysis from 50 to 99°C (15 s/10 s). For *Roseobacter*-quantification, a two-step qPCR was performed as described above with the same time intervals, but different annealing temperatures (annealing at 66°C for 5 cycles and 63°C for 50 cycles). All samples were analyzed in four independent runs with triplicates, each. Quantification standards were produced as described by Süß et al. (2004) using genomic DNA of *Dinoroseobacter shibae* as template. The resulting 16S rRNA gene amplicons were purified by the QIAquick PCR purification kit according to the manufacturer's instructions (Qiagen, Germany).

### Next-generation sequencing

The bacterial diversity was analyzed by next-generation sequencing. While the total bacterial community was assessed by 16S rRNA gene sequencing, the potentially active fraction was determined by an RNA-based approach analyzing 16S rRNA transcripts. For RNA analysis, first a DNAse digestion and purification was performed as described by Schneider et al. (2017) and cDNA was generated from DNA-free RNA by SuperScript III (Thermo Fisher Scientific, USA) reverse transcription as described by Wemheuer et al. (2015) using the reverse primer S-D-Bact-0785-a-A-21 without MiSeq adapter (5′-GAC TAC HVG GGT ATC TAA TCC-3′; Klindworth et al., 2013). Extracted DNA was treated with RNAse A as described by Schneider et al. (2017) and purified using the GeneRead Size Selection kit (Qiagen, Germany) according to manufacturer's instructions with one modification: samples were eluted in two steps, first using DEPC-treated water and subsequently elution buffer (supplied by the kit).

16S rRNA amplicon libraries were generated from DNA and cDNA by PCR using Phusion Polymerase as described by Wemheuer et al. (2015) with the forward primer S-D-Bact-0341-b-S-17 (5′-CCT ACG GGN GGC WGC AG-3′) and the reverse primer S-D-Bact-0785-a-A-21 (5′-GAC TAC HVG GGT ATC TAA TCC-3′; both Klindworth et al., 2013) with Illumina Nextera adapters for sequencing. Each sample was subjected to three independent amplifications and pooled in equal amounts. Amplification products of cDNA were purified using NucleoMag NGS Clean-up and Size Select (Macherey-Nagel, Germany). Both, DNA and cDNA samples were quantified with a NanoDrop ND-1000 spectrophotometer (Thermo Fisher Scientific, USA) and barcoded using the Nextera XT-Index kit (Illumina, USA) and the Kapa HIFI Hot Start polymerase (Kapa Biosystems, USA). Sequencing was performed at the Goettingen Genomics Laboratory on an Illumina MiSeq System (paired end 2 × 300 bp) using the MiSeq Reagent Kit v3 (Illumina, USA).

### Processing and analysis of illumina-datasets

Generated datasets of 16S rRNA genes and transcripts were processed according to Granzow et al. (2017). The Trimmomatic version 0.32 (Bolger et al., 2014) was initially used to truncate low quality reads if quality dropped below 20 in a sliding window of 10 bp. Datasets were subsequently processed with Usearch version 8.0.1623 (Edgar, 2010) as described in Wemheuer and Wemheuer (2017). In brief, paired-end reads were merged and quality-filtered. Filtering included the removal of low quality reads (maximum number of expected errors >1 and more than 1 ambitious base, respectively) and those shorter than 200 bp. Processed sequences of all samples were joined and clustered in operational taxonomic units (OTUs) at 3% genetic divergence using the UPARSE algorithm implemented in Usearch. A *de novo* chimera removal was included in the clustering step. All OTUs consisting of one single sequence (singletons) were removed. Afterwards, remaining chimeric sequences were removed using the Uchime algorithm in reference mode with the most recent RDP training set (version 15) as reference dataset (Cole et al., 2009). Afterwards, OTU sequences were taxonomically classified using QIIME (Caporaso et al., 2010) by BLAST alignment against the SILVA database (SILVA SSURef 128 NR) and the QIIME release of the UNITE database (version 7.1; August 2016), respectively. All non-bacterial OTUs were removed based on their taxonomic classification in the respective database. Subsequently, processed sequences were mapped on OTU sequences to calculate the distribution and abundance of each OTU in every sample. Sequence data were deposited in the sequence read archive (SRA) of the National Center for Biotechnology Information (NCBI) under the accession numbers SRR5740175—SRR5740185 and SRR5740189—SRR5740196 (BioProject PRJNA391422). Details can be found in the Table S1.

### Phylogenetic analysis

To display the next relatives of the OTUs identified as “uncultured,” the consensus sequences of these OTUs were aligned with the integrated aligner of ARB (version 6.0.2; Ludwig et al., 2004). Afterwards the sequences were added to the SILVA-backbone tree (SSURef NR99 128) by the maximum-parsimony method using the Quick-Add function in ARB. Genera outside the family *Rhodobacteraceae* and the genera which were not related to the defined OTUs were removed to simplify the tree. Fifty sequences of *Rhizobium sp*. served as root.

### Statistical data analysis

Statistical analyses were performed in R (version 3.4.0; R Core Team, 2015). Differences were considered statistically significant with *p* ≤ 0.05. Environmental data (including concentrations of TOC, S, NO_3_, NH_4_, PO_4_, silicic acid, Fe_2_O_3_, and MnO_2_ as well as chlorophyll concentrations) were normalized using the “scale” function in R. Non-metric multidimensional scaling (NMDS) was done with GUniFrac (version 1.0; Chen et al., 2012) and weight parameter α = 0. A rooted phylogenetic tree for the analysis was generated with muscle (version 3.8.31). Environmental fit was calculated using the vegan package (version 2.4-3; Oksanen et al., 2017). The NMDS plots were generated with the standard plot function in R. Significant environmental factors (*p* ≤ 0.05) were plotted onto the graph as arrows. Members of the *Roseobacter* group as well as the oceanic provinces were clustered by similarity in standard R heat maps. All files and scripts used to generate the plots can be found in the supplements (data sheet 1). Image editing was done in inkscape (version 0.91).

## Results

### Nutrient concentrations increase along the transect toward the northern pacific

**Figure 2:**
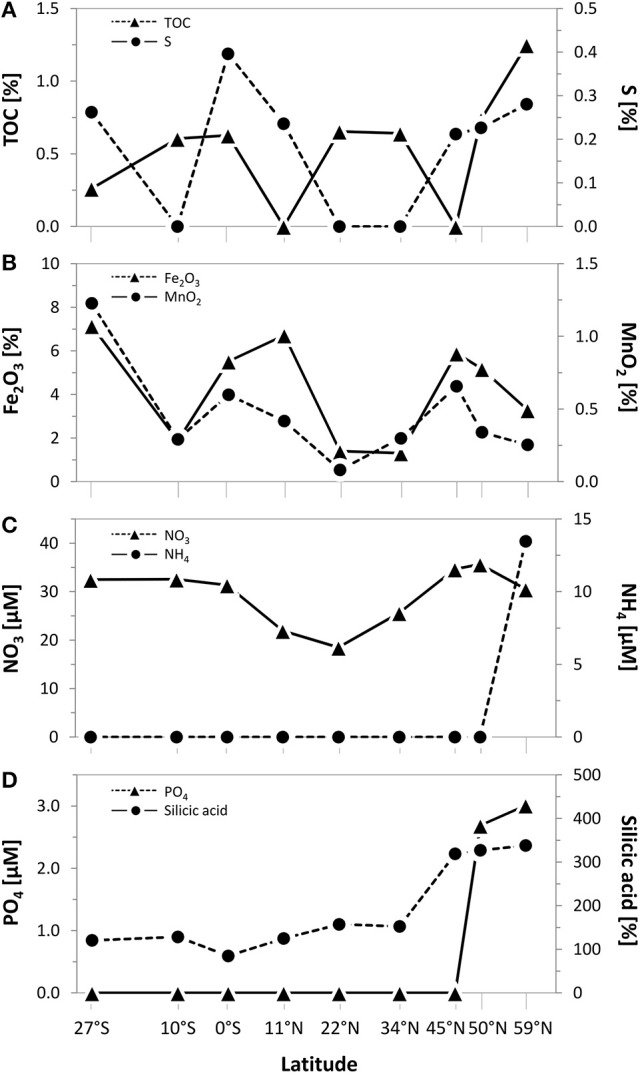
Geochemical composition of surface sediments (0–1 cmbsf) along the Pacific transect from 27°S to 59°N. Concentrations of **(A)** total organic carbon (TOC) and sulfur as well as **(B)** iron(III) oxide and manganese dioxide in bulk sediments. Porewater concentrations of **(C)** nitrate and ammonium as well as **(D)** phosphate and silicic acid. Values plotted as 0 were below quantification limit.

Biogeographical oceanic provinces as defined by Longhurst (2007) are characteristic by varying nutrient regimes. The chlorophyll concentrations (summed over the upper 500 m; Table 1), reflecting the activity in the water column, were lowest at the sampling sites associated to both Pacific gyres (27°S and 22°N), while the amount of chlorophyll was twice as high at the equator (102 mg/m^3^). At the northernmost sites located in the Pacific subarctic region and the Bering Sea highest amounts were found (approximately 120 mg/m^3^). Within the sediments, the total organic carbon (TOC) content as a general indicator for nutrient availability fluctuated around 0.5% of the sediment dry weight, showing an opposing trend with sulfur at most sites of the transect. A maximum TOC content of 1.3% was found in the Bering Sea at 59°N (Figure 2A). Iron(III) oxide and manganese dioxide showed an equal trend and were increased at the southernmost site, the equator and within the Pacific subarctic region (Figure 2B). Nitrate concentrations in porewaters fluctuated around 30 μM along the transect, showing a maximum of approximately 35 μM at the sampling sites located in the Pacific subarctic region (45°N and 50°N; Figure 2C). Concentrations of ammonia and phosphate were 13.5 μM and 2.7–3.0 μM at the northernmost sites, while the concentrations were below quantification limit at all other sites of the transect. Silicic acid was relatively constant at a level of approximately 130 μM from 27°S to 34°N, followed by an increase to >310 μM in the Pacific subarctic region and remained at this high level further north (Figure 2D). In general, most parameters showed an increasing trend toward the north with highest concentrations in the Pacific subarctic region and the Bering Sea.

### Total cell numbers at the seafloor correlate to increasing nutrient concentrations

**Figure 3:**
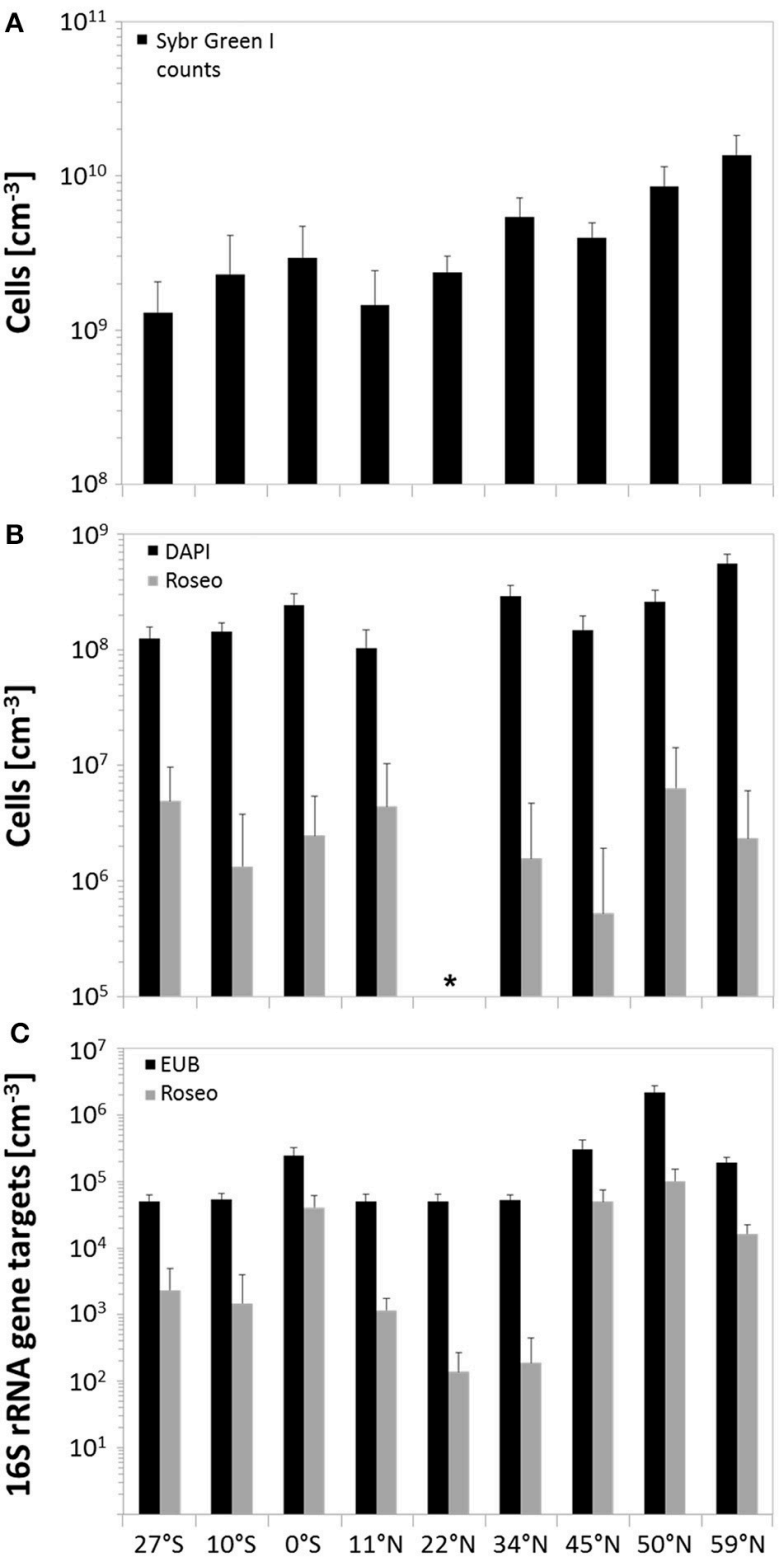
Microbial abundance in sediments along the Pacific transect, arranged from south to north. **(A)** Total cell counts by using SYBR Green I staining; **(B)** quantification of benthic members of the *Roseobacter* group by CARD-FISH and associated DAPI counts; **(C)** amount of 16S rRNA gene targets of bacteria and the *Roseobacter* group determined by qPCR. Error bars indicate standard deviations of the arithmetic mean. ^*^CARD-FISH analysis of site 22°N could not be performed due to strong background fluorescence of the sediment matrix.

In general, SYBR Green I counting revealed total cell numbers of 1.3 × 10^9^ to 1.4 × 10^10^ cells × cm^−3^ of sediment (Figure 3A). Lowest cell numbers were counted at the edge of the south Pacific gyre and within the north Pacific gyre (27°S and 11°N) with a slight increase at the equator. Cell numbers increased further north, showing maximum values in the highly productive Bering Sea. All DAPI counts (Figure 3B) that were obtained from counterstaining of the CARD-FISH filters were approximately one order of magnitude lower than the SYBR Green I counts. Even though the variations along the transect were less pronounced, both techniques showed a comparable trend. While lowest DAPI counts were also found at 27°S and 11°N (1.0–1.3 × 10^8^ cells × cm^−3^), a maximum was detected in the Bering Sea (5.6 × 10^8^ cells × cm^−3^). Molecular quantification by qPCR revealed even lower numbers ranging between 10^4^ and 10^6^ bacterial 16S rRNA gene targets per cm^3^ of sediment. But, independent of the quantification method, the counts correlated to the nutrient concentrations within the sediments and the productivity of the overlying water column with increasing numbers toward the Bering Sea.

### Relative and direct quantification of the *Roseobacter* group indicates their contribution to the total bacterial communities

The relative abundance of the *Roseobacter* group within the total bacterial community can be estimated from the results of next-generation sequencing. While Illumina sequencing of 16S rRNA genes was used to determine the DNA-based bacterial diversity, the active community was identified by an RNA-based approach targeting 16S rRNA transcripts. Sequencing resulted in a total number of 382,651 sequences over all sampling sites affiliated to 4455 different OTUs (at 3% genetic divergence). Following decreasing phylogenetic levels, *Alphaproteobacteria* accounted for 5% (59°N) to 19% (22°N) of the DNA-based bacterial community and even up to 53% of the active community at the edge of the south Pacific gyre (27°S). The family *Rhodobacteraceae*, mainly composed of the *Roseobacter* group, contributed up to 1.3% of the DNA-based and 3% of the RNA-based bacterial community (both 45°N). As only one *Paracoccus*-affiliated OTU was detected and the OTU identified as “uncultured V” was probably affiliated to the genus *Halovulum* (Figure S1), the *Roseobacter* group (including all other uncultured representatives) made up similar proportions. It constituted 0.3 to 1.3% of the DNA-based and 0.1 to 3% of the RNA-based community (Table 2). Interestingly, the active community contained a higher proportion of *Roseobacter* group members (average: 0.9%) in comparison to the DNA-based community (average: 0.7%).

**Table 2:** Proportion of *Roseobacter*-affiliated OTUs on the total bacterial communities along the Pacific transect.

**Site**	**DNA-based community (16S rRNA genes)**			**RNA-based community (16S rRNA transcripts)**		
	**Total OTUs**	***Roseobacter*****-affiliated OTUs**		**Total OTUs**	***Roseobacter*****-affiliated OTUs**	
		**No**.	**%**		**No**.	**%**
27°S	25740	69	0.27	21345	58	0.27
10°S	26955	170	0.63	20729	5	0.02
0°S	18856	200	1.06	26743	393	1.47
11°N	22665	102	0.45	25985	99	0.38
22°N	22954	95	0.41	29070	18	0.06
34°N	26421	137	0.52	14637	20	0.14
45°N	24009	318	1.32	22071	662	3.00
50°N	15695	132	0.84	18584	291	1.57
59°N	11025	66	0.60	9167	102	1.11

*The complete dataset contained 382,651 OTUs*.

As Illumina sequencing only results in estimates of relative numbers, direct quantification of the *Roseobacter* group was performed. Microscopic quantification using a *Roseobacter*-specific CARD-FISH probe revealed an average of 3 × 10^6^ cells × cm^−3^ of sediment, corresponding to a relative abundance of 1.7%. At 11°N, up to 4.3% of all DAPI counts were affiliated to the *Roseobacter* group, while at 45°N this group only made up a proportion of 0.4% (Figure 3B). Molecular analysis by *Roseobacter*-specific qPCR mostly exhibited lower values compared to CARD-FISH quantification. Quantitative PCR revealed 10^2^ to 10^5^ 16S rRNA gene targets per cm^3^ of sediment along the transect, showing highest values at the equator and 45°N (Figure 3C). On average, *Roseobacter* gene targets accounted for 6.3% of all bacterial 16S rRNA genes.

### The community composition of the *Roseobacter* group is dominated by uncultured representatives

**Figure 4:**
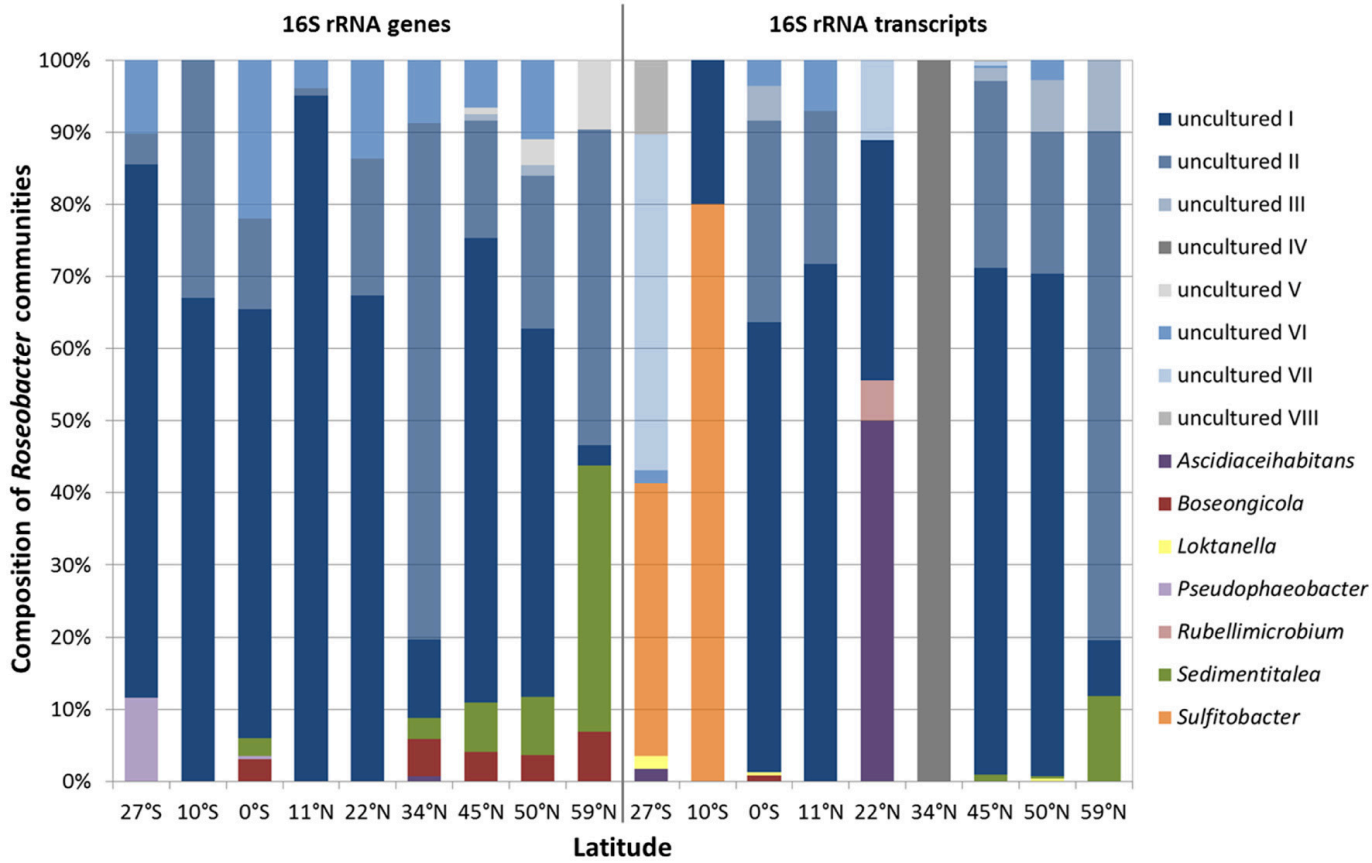
Relative abundance of single *Roseobacter*-affiliated OTUs on the community composition of the *Roseobacter* group within Pacific surface sediments. Displayed are the DNA-based communities, identified by 16S rRNA gene sequencing (left panel) and the active communities, derived from the 16S rRNA transcript library (right panel).

Each sampling site was characterized by individual community patterns. Remarkably, out of the 15 different *Roseobacter*-affiliated OTUs, eight of them could not be assigned to known genera and were classified as “uncultured” during the processing of the Illumina dataset (Figure 4, blue bars “uncultured I-VIII”). Phylogenetical analysis (Figure S1) showed that the OTUs identified as “uncultured I-III” interestingly clustered with each other and did not show any relation to known genera within the family *Rhodobacteraceae*. This indicates the lack of isolates and the amount of unexplored diversity within the *Roseobacter* group. Notably, the OTU assigned to “uncultured I” made up the highest proportion within the DNA-based as well as in the RNA-based community (Figure 4). The next cultured relatives to the OTU “uncultured IV” were within the genus *Loktanella*, while the OTU assigned to “uncultured VI” clustered with the genus *Pacifibacter*. The OTUs affiliated to “uncultured VII and VIII” were related to the genera *Litorimicrobium* and *Ruegeria*, respectively. The OTU classified as “uncultured V” was distantly related to the genus *Halovulum*, a member of the *Amaricoccus* group (Figure S1).

Focusing on the relative abundance of known genera that contribute to the diversity of the *Roseobacter* group within the DNA-based community, especially the genera *Sedimentitalea* and *Boseongicola* were found (Figure 4 left panel). Most genera were broadly spread across the transect, however *Pseudophaeobacter* was only detected at the equator and further south. Within the active community, *Loktanella* and *Sedimentitalea* were distributed over one third of all sampling sites, while the latter together with *Sulfitobacter* made up the largest proportions of benthic *Roseobacter* group members with cultured representatives (Figure 4 right panel).

### Oceanic provinces that differ in primary production and nutrient availability within the sediments exhibit distinct communities of benthic members of the *Roseobacter* group

**Figure 5:**
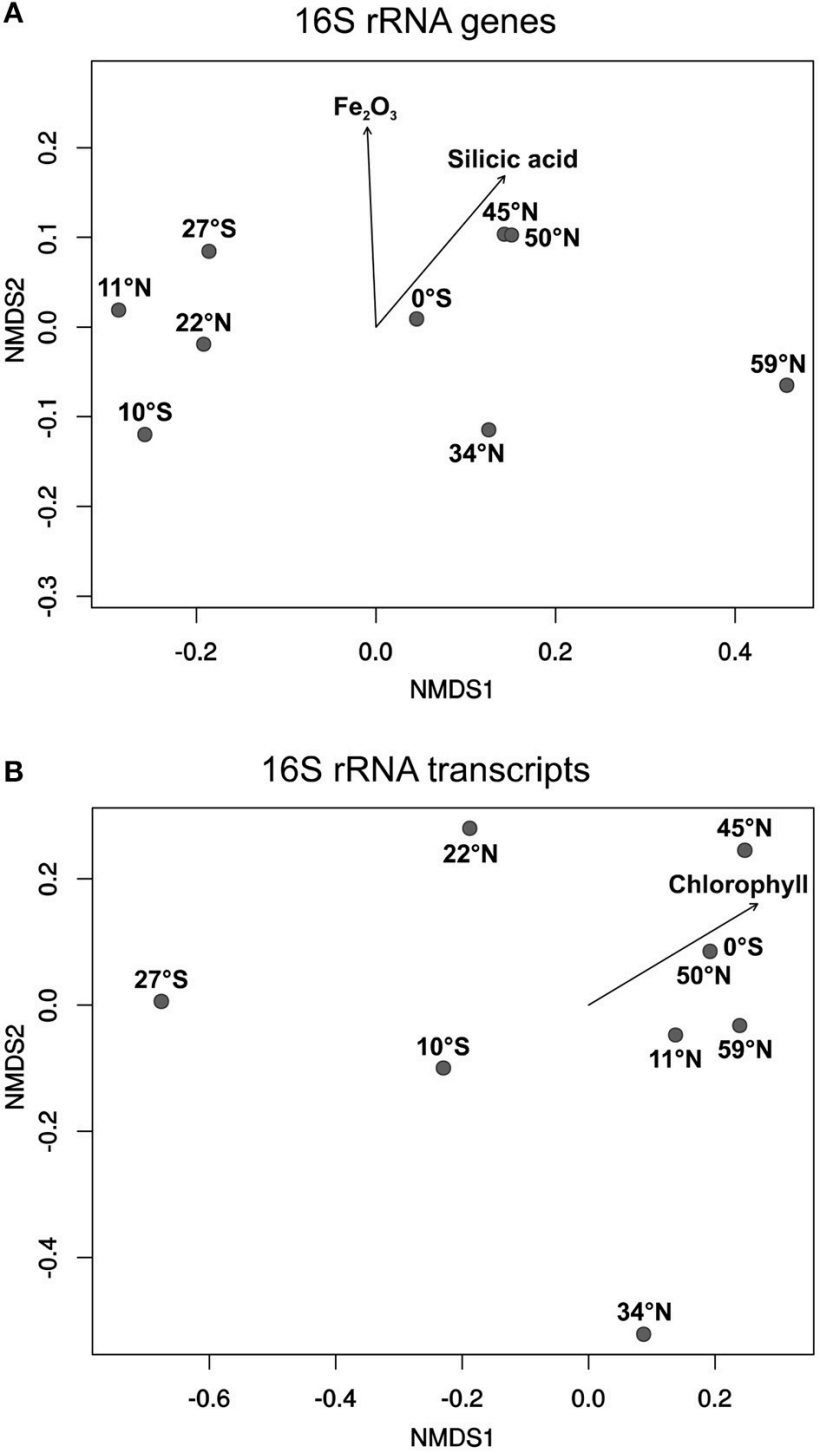
Non-metric multidimensional scaling (NMDS) plots of **(A)** the DNA-based and **(B)** the RNA-based community compositions of the *Roseobacter* group. Plots were calculated from 16S rRNA gene and 16S rRNA transcript libraries. Arrows indicate the relation of the *Roseobacter* group to significant environmental factors (*p* ≤ 0.05).

To express the specific distribution patterns, non-metric multidimensional scaling (NMDS) was performed to compare the community composition at the different sampling sites to the respective environmental parameters. The DNA-based communities of benthic members of the *Roseobacter* group at the various sampling sites clearly clustered separately depending on local nutrient availability in the sediments, and on the primary production in the water column (Figure 5A). Sampling sites from both oligotrophic Pacific gyres (27°S, 10°S, 11°N, 22°N) formed one cluster, while the equator, characterized by higher nutrient content in the sediment and the water column, clustered with all other more eutrophic sampling sites. A most distinct community composition was found at the northernmost sampling site in the Bering Sea, exhibiting elevated nutrient concentrations. The iron(III) oxide content of the sediment as well as the concentrations of silicic acid in the porewater were found to significantly influence the community composition of benthic members of the *Roseobacter* group (*p* ≤ 0.05). The varying high MnO_2_ content of the deep-sea sediments indicate oxic environmental conditions at all sites and possibly a hydrothermal contribution at some sites. Surprisingly, this parameter had no influence on the community composition.

All active, RNA-based, communities of the *Roseobacter* group generally showed a less uniform composition compared to the DNA-based communities (Figure 5B). Apart from the sampling site at 11°N, the other three oligotrophic sites (27°S, 10°S, 22°N) were also separated from the more eutrophic regions. Here, site 34°N was more distinct as it was exclusively composed of the OTU identified as “uncultured IV” (phylogenetically assigned to the genus *Loktanella*). Interestingly, only the chlorophyll content of the overlying water column was identified as a significant environmental control on the diversity of active *Roseobacter* group members.

### The oligotrophic gyres and the more eutrophic regions are distinguished by different genera within the DNA- and RNA-based fraction of the *Roseobacter* group communities

**Figure 6:**
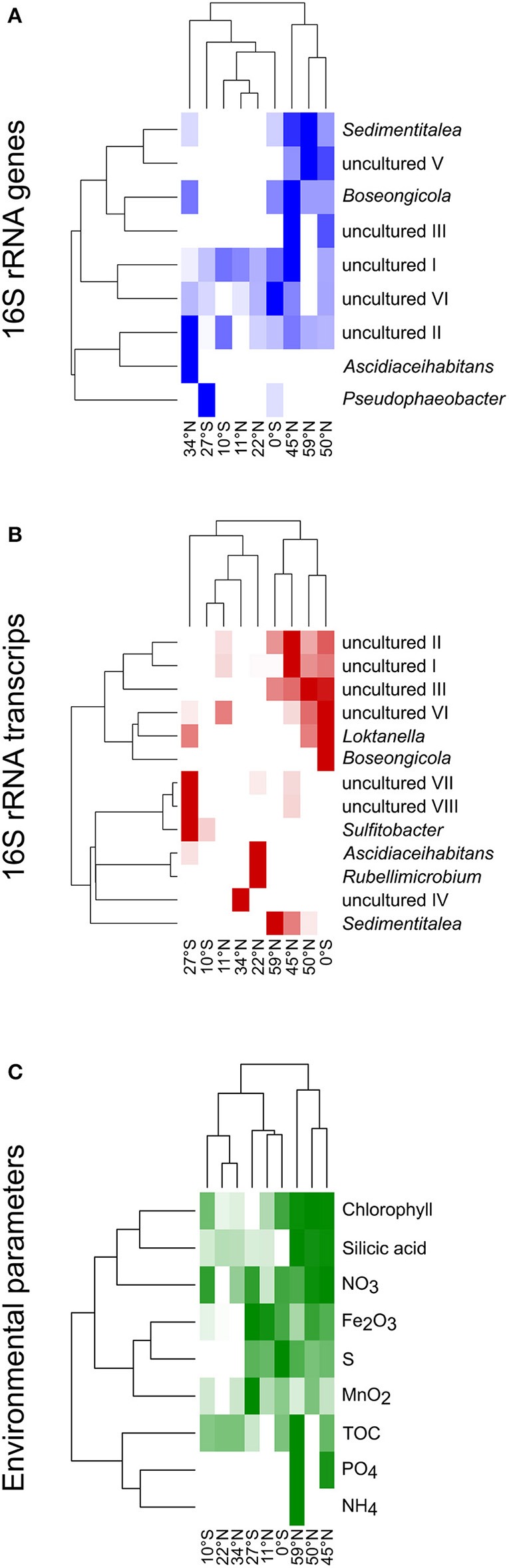
Heat maps for the different sampling sites according to the compositions of **(A)** the DNA-based, **(B)** the RNA-based community composition of the *Roseobacter* group and **(C)** nutrients in sediments and porewaters as well as chlorophyll in the water column. The clustering on the left side reflects the relative frequencies of the different OTUs/nutrient concentrations, while on top the clustering based on the similarity of the sampling sites is shown.

Heat maps were calculated for a more detailed view on the contribution of single genera to the community composition of benthic members of the *Roseobacter* group at the different sampling sites (Figure 6). Both, the DNA- and RNA-based communities of the sites in the North Pacific gyre (11°N and 22°N) clustered together with the site at 10°S, while the northernmost sites (45°N, 50°N, 59°N) formed another cluster that branched separately. In contrast, the equator and the sites at the edge of the south Pacific gyre (27°S) and at the north Pacific polar front varied in their assignment. In the DNA-based communities of the *Roseobacter* group, the branching of the northernmost sites was mainly due to the presence of the OTUs affiliated to *Sedimentitalea, Boseongicola* and “uncultured V” (Figure 6A). The DNA-based communities of the North Pacific gyre and the site at 10°S were in turn characterized by OTUs assigned to “uncultured I and II,” the OTU “uncultured VI” (phylogenetically affiliated to *Pacifibacter*) and the absence of other OTUs. *Ascidiaceihabitans* and *Pseudophaeobacter*-affiliated OTUs only contributed to the DNA-based community composition of the varying sites at 27°S, 0°S, and 34°N.

The active community compositions were generally more diverse than those of the DNA-based communities (Figure 6B). Here, the equatorial community branched with the communities of the northernmost sites. While all of these sites were characterized by OTUs assigned to some uncultured representatives (“uncultured I-III and VI”), *Sedimentitalea* and *Boseongicola*-affiliated OTUs were only found at the three northernmost sites and the equator, respectively. Branching of both Pacific gyres and site 34°N was mainly due to the presence and absence of single OTUs (e.g., *Rubellimicrobium, Sulfitobacter, Ascidiaceihabitans* or “uncultured IV”, phylogenetically affiliated to *Loktanella*).

The heat map for single environmental parameters in relation to the sampling sites showed a slightly different branching of the investigated sites (Figure 6C). Here, the sites at 10°S and 22°N clustered with the site located at the edge of the north Pacific polar front due to low concentrations of all nutrients analyzed. The northernmost sites branched together, but separate of all other sites, as the Pacific subarctic region and the Bering Sea are characterized by elevated nutrient concentrations.

## Discussion

### Microbial abundance within the sediments is related to nutrient availability and primary production in the different oceanic provinces

Organic matter input and quality, but also water depth and marine productivity in the water column, influence the cell abundance within sediments. Distinct oceanic provinces were crossed along the Pacific transect that are characterized by varying primary production, resulting in changing nutrient availability at the seafloor (Longhurst, 2007). The *in situ* chlorophyll measurements (summed over the upper 500 m) were higher compared to the concentrations determined by satellite imaging for the time period of the expedition (Figure 1) as the satellite only detects surface chlorophyll. The results of the satellite imaging are very useful for the classification of the oceanic provinces, but the *in situ* measurements include the deep chlorophyll maximum and thus reflect the total primary production.

Variations in benthic cell numbers depend on the primary production in the water column and are strongly correlated to sedimentation rates and distance from land (Kallmeyer et al., 2012). In general, total cell numbers determined by SYBR Green I counting of around 10^9^ cells × cm^−3^ were in the expected range for seafloor sediments (Parkes et al., 2000; D'Hondt et al., 2004). DAPI counts performed as counterstaining for CARD-FISH quantification showed the same trend, but were approximately one magnitude lower than SYBR Green I counts as already observed by Weinbauer et al. (1998) and Morono et al. (2009). Furthermore, CARD-FISH quantification, targeting the RNA, might be influenced by low numbers of active cells or unspecific fluorescence of the sediment matrix, as e.g., observed for the site located at 22°N. This bias can partially be compensated by other quantification attempts such as qPCR or identifying the relative amount of specific OTUs in the Illumina sequence libraries. While qPCR significantly depends on the DNA extraction efficiency and might underestimate total numbers (e.g., sites at 0°N and 45°N), the results of next-generation sequencing should be interpreted as relative values and not as absolute numbers. Thus, each method should be evaluated separately, but trends in abundances may be confirmed (Lloyd et al., 2013).

All quantification methods used to estimate the total amount of bacteria showed a general trend following the primary production in the different oceanic provinces and the nutrient availability within the sediments. Cell numbers were lowest in sediments of the north Pacific gyre and at the edge of the south Pacific gyre. Both provinces are characterized by low primary production and limiting nutrient concentrations, e.g. nitrate content (Rullkötter, 2006; Longhurst, 2007), resulting in extremely low sedimentation rates (Røy et al., 2012). By all general quantification methods, we confirmed that the upwelling along the equator leads to higher cell numbers at the seafloor due to elevated primary production in the water column. The more pronounced increase in bacterial abundance from the edge of the north Pacific polar front to the Bering Sea also follows the productivity in the water column. The highest cell numbers, which were found in the Bering Sea, are presumably due to the proximity to land in combination with the coastal runoff, leading to increased phytoplankton activities in the water column (Longhurst, 2007; Kallmeyer et al., 2012). The high primary production causes high sedimentation rates and therefore elevated nutrient concentrations at the seafloor (Schulz and Zabel, 2006; Wehrmann et al., 2011). The measured TOC content of the sediments, for instance, was around 1% as also reported by Seiter et al. (2004). In general, as all other nutrient concentrations increased in the northernmost sites, total cell counts followed this trend.

### Pacific sediments show similar maximum proportions of the *Roseobacter* group as coastal areas, but exhibit lower average abundances

As all investigated sampling sites of the Pacific transect exhibited water depths of several thousand meters, organic matter reaching the seafloor is supposed to be more recalcitrant due to microbial degradation while sinking (Martin et al., 1987; Karl et al., 1988). Although only 1% of the primary production reaches the seafloor, 97% of this material is decomposed by microbial activities and returned as dissolved matter to the water column (Zabel and Hensen, 2006). Interestingly, chlorophyll was identified as significant environmental control on the diversity of active members of the *Roseobacter* group (Figure 5B). This proofs that not only the nutrient availability in the sediment alone is triggering the distribution of different benthic *Roseobacter* genera, but also the productivity in the water column has major influence.

The fact that the average abundance of *Roseobacter*-affiliated OTUs in our dataset was much lower than the maximum proportions is probably due to the oligotrophic nature of the investigated oceanic provinces. Furthermore, while the Pacific transect includes deep-sea sediments distinct from landmasses, exhibiting low nutrient contents, most other benthic studies were performed in coastal, nutrient-rich sediments (Buchan et al., 2005). However, the relative amount of *Roseobacter*-affiliated OTUs in our 16S rRNA transcript library of up to 3% is comparable to proportions found in coastal sediments from the North Sea and cold seeps in the Nankai Trough, both around 2% of all 16S rRNA genes (Li et al., 1999; Kanukollu et al., 2016), as well as in volcanic sediments of the Sea of Okhotsk (Inagaki et al., 2003) and Antarctic Shelf sediments (Bowman and McCuaig, 2003). Differences in abundance between coastal and open ocean sites are not only visible at the seafloor. Even in the water column of the North Pacific, the *Roseobacter* group only made up 5% of the community associated to phytoplankton blooms (Tada et al., 2011), representing a lower abundance as described in previous studies on coastal regions (González et al., 2000; Selje et al., 2004; Giebel et al., 2009). The maximum amount of *Roseobacter* group members, as quantified by CARD-FISH (up to 4.3%), is within the range that was previously reported for tidal-flat sediments by Lenk et al. (2012). The proportion of *Roseobacter* group members quantified by qPCR with an average of 6.3% is again in the same range. However, the very high percentages of approximately 16% at sites 0°N and 45°N might be due to an underestimation of the total abundance by the bacteria-specific qPCR.

### The dominance of uncultured members of the *Roseobacter* group in sediments hampers to directly link their function to environmental settings

Almost all of the detected genera that have cultivated representatives were described as aerobic heterotrophs and were previously isolated from coastal marine sediments. For instance, *Sedimentitalea*, which is found in both, the DNA- and RNA-based communities, was originally isolated from sandy sediments of the South China Sea (Sun et al., 2010; Breider et al., 2014). The type strain of *Boseongicola* derived from tidal-flat sediments (Park et al., 2014) and *Loktanella* from a deep subseafloor sediment off the coast of Japan (Van Trappen et al., 2004; Tsubouchi et al., 2013). While *Loktanella* and *Sulfitobacter*-affiliated OTUs were found in coastal North Sea sediments (Kanukollu et al., 2016), none of the above mentioned genera were previously detected in deep-sea sediments of open ocean sites. The presence of *Loktanella* and *Sulfitobacter* in coastal sediments may suggest that they depend on high nutrient concentrations. However, our results show that they also constitute to the community of active *Roseobacter* group members in oligotrophic open-ocean environments.

The Illumina datasets of benthic members of the *Roseobacter* group were composed of OTUs affiliated to the above mentioned species, but mainly of OTUs assigned to “uncultured” (84% on average). The consensus sequences of these OTUs of around 440 bp length, allowed a phylogenetic classification on family level, but the exact affiliation to a specific genus still remains unclear. For a fixed assignment, the complete 16S rRNA sequence or even an isolate is needed. Nevertheless, the database entries of the sequence branching together with OTUs assigned to “uncultured I to III” reveal “Pacific Ocean sediments” as isolation source (e.g., accession numbers JX227630, AJ567557; JX226780, EU491756; KM071672; Figure S1). The large proportion of *Roseobacter*-affiliated OTUs in both sequence libraries that are classified as “uncultured” indicates the presence of a specific and active community of benthic members of the *Roseobacter* group that is well adapted to conditions at the seafloor of the deep sea. These bacteria were not yet cultured as they might grow very slow, form microcolonies or depend on the co-occurrence of other microorganisms. Furthermore, this proves the assumption that the environmental conditions can hardly be mimicked in the laboratory (Amann et al., 1995; Kaeberlein et al., 2002). Due to a lack of isolates, their physiological characteristics are largely unknown and hamper further interpretation concerning their metabolism in sediments. However, as we have now correlated the environmental settings to the abundance and co-occurrence of single OTUs, specific media for the isolation of so-far uncultured benthic members of the *Roseobacter* group can be designed. Furthermore, the sequence information allows developing primers and probes for the detection and quantification of the different uncultured *Roseobacter* representatives in enrichment cultures and environmental samples.

## Author contributions

MP, JD, and BE performed the sediment sampling during RV Sonne expedition SO248. JD did the molecular quantification (cell counting, CARD-FISH, qPCR) and extraction of nucleic acids. NE did the nutrient measurements, supervised and interpreted by BS. AvH-H prepared samples for Illumina sequencing and did statistical analyses. BW processed the Illumina sequence data. TB collected and provided chlorophyll data. MP and BE wrote the first draft of the manuscript including figure design. All authors contributed were involved in critical revision and approval of the final version.

### Conflict of interest statement

The authors declare that the research was conducted in the absence of any commercial or financial relationships that could be construed as a potential conflict of interest.
