# Supplementary material for: The Biogeographical Distribution of Benthic Roseobacter Group Members along a Pacific Transect Is Structured by Nutrient Availability within the Sediments and Primary Production in Different Oceanic Provinces
Source: Front Microbiol. 2017 Dec 18;8:2550. doi: 10.3389/fmicb.2017.02550 (PMC5741685; doi:10.3389/fmicb.2017.02550)
Supplement: Supplementary file 1 [file Table1.PDF]

Table S1: Details of NCBI submission

Site	Description	Run accession	Sample name	Release date	Load date	Spots	Bases	Spots_with_mates	Average length	Size_MB	Download path	Experiment	SRA study	BioProject	ProjectID	Sample	BioSample	TaxID	Scientific name	Submission
27°S	16S rRNA transcript analysis	SRR5740175	SO248_St2_0-1cm	15.11.2017 23:01	22.06.2017 09:04	49675	26784599	49675	539	13	https://sra-download.ncbi.nlm.nih.gov/traces/sra49/SRR/005605/SRR5740175	SRX2946080	SRP110130	PRJNA391422	391422	SRS2305523	SAMN07268532	412755	marine sediment metagenome	SRA579333
10°S	16S rRNA transcript analysis	SRR5740176	SO248_St4_0-1cm	15.11.2017 23:01	22.06.2017 09:04	39750	23106865	39750	581	12	https://sra-download.ncbi.nlm.nih.gov/traces/sra49/SRR/005605/SRR5740176	SRX2946079	SRP110130	PRJNA391422	391422	SRS2305522	SAMN07268533	412755	marine sediment metagenome	SRA579333
22°N	16S rRNA gene analysis	SRR5740177	SO248_St10_0-1cm	15.11.2017 23:01	22.06.2017 09:04	43822	26039163	43822	594	13	https://sra-download.ncbi.nlm.nih.gov/traces/sra49/SRR/005605/SRR5740177	SRX2946078	SRP110130	PRJNA391422	391422	SRS2305512	SAMN07268536	412755	marine sediment metagenome	SRA579333
34°N	16S rRNA gene analysis	SRR5740178	SO248_St12_0-1cm	15.11.2017 23:01	22.06.2017 09:04	51088	30406708	51088	595	16	https://sra-download.ncbi.nlm.nih.gov/traces/sra49/SRR/005605/SRR5740178	SRX2946077	SRP110130	PRJNA391422	391422	SRS2305514	SAMN07268537	412755	marine sediment metagenome	SRA579333
45°N	16S rRNA gene analysis	SRR5740179	SO248_St14_0-1cm	15.11.2017 23:01	22.06.2017 09:04	47681	28517496	47681	598	14	https://sra-download.ncbi.nlm.nih.gov/traces/sra49/SRR/005605/SRR5740179	SRX2946076	SRP110130	PRJNA391422	391422	SRS2305518	SAMN07268538	412755	marine sediment metagenome	SRA579333
50°N	16S rRNA gene analysis	SRR5740180	SO248_St16_0-1cm	15.11.2017 23:01	22.06.2017 09:04	55086	33030399	55086	599	17	https://sra-download.ncbi.nlm.nih.gov/traces/sra49/SRR/005605/SRR5740180	SRX2946075	SRP110130	PRJNA391422	391422	SRS2305519	SAMN07268539	412755	marine sediment metagenome	SRA579333
27°S	16S rRNA gene analysis	SRR5740181	SO248_St2_0-1cm	15.11.2017 23:01	22.06.2017 09:04	48997	29112497	48997	594	15	https://sra-download.ncbi.nlm.nih.gov/traces/sra49/SRR/005605/SRR5740181	SRX2946074	SRP110130	PRJNA391422	391422	SRS2305523	SAMN07268532	412755	marine sediment metagenome	SRA579333
10°S	16S rRNA gene analysis	SRR5740182	SO248_St4_0-1cm	15.11.2017 23:01	22.06.2017 09:04	48853	28964390	48853	592	15	https://sra-download.ncbi.nlm.nih.gov/traces/sra49/SRR/005605/SRR5740182	SRX2946073	SRP110130	PRJNA391422	391422	SRS2305522	SAMN07268533	412755	marine sediment metagenome	SRA579333
0°S	16S rRNA gene analysis	SRR5740183	SO248_St6_0-1cm	15.11.2017 23:01	22.06.2017 09:04	57607	34459424	57607	598	18	https://sra-download.ncbi.nlm.nih.gov/ traces/sra49/SRR/005605/SRR5740183	SRX2946072	SRP110130	PRJNA391422	391422	SRS2305513	SAMN07268534	412755	marine sediment metagenome	SRA579333
11°N	16S rRNA gene analysis	SRR5740184	SO248_St8_0-1cm	15.11.2017 23:01	22.06.2017 09:04	50442	30198382	50442	598	15	https://sra-download.ncbi.nlm.nih.gov/traces/sra49/SRR/005605/SRR5740184	SRX2946071	SRP110130	PRJNA391422	391422	SRS2305515	SAMN07268535	412755	marine sediment metagenome	SRA579333
59°N	16S rRNA gene analysis	SRR5740185	SO248_St19_0-1cm	15.11.2017 23:01	22.06.2017 09:04	41693	24969377	41693	598	13	https://sra-download.ncbi.nlm.nih.gov/traces/sra49/SRR/005605/SRR5740185	SRX2946070	SRP110130	PRJNA391422	391422	SRS2305516	SAMN07268540	412755	marine sediment metagenome	SRA579333
50°N	16S rRNA transcript analysis	SRR5740189	SO248_St16_0-1cm	15.11.2017 23:01	22.06.2017 09:04	47205	28025524	47205	593	14	https://sra-download.ncbi.nlm.nih.gov/traces/sra49/SRR/005605/SRR5740189	SRX2946066	SRP110130	PRJNA391422	391422	SRS2305519	SAMN07268539	412755	marine sediment metagenome	SRA579333
45°N	16S rRNA transcript analysis	SRR5740190	SO248_St14_0-1cm	15.11.2017 23:01	22.06.2017 09:04	46928	27523159	46928	586	14	https://sra-download.ncbi.nlm.nih.gov/traces/sra49/SRR/005605/SRR5740190	SRX2946065	SRP110130	PRJNA391422	391422	SRS2305518	SAMN07268538	412755	marine sediment metagenome	SRA579333
59°N	16S rRNA transcript analysis	SRR5740192	SO248_St19_0-1cm	15.11.2017 23:01	22.06.2017 09:04	41779	24311022	41779	581	12	https://sra-download.ncbi.nlm.nih.gov/traces/sra49/SRR/005605/SRR5740192	SRX2946063	SRP110130	PRJNA391422	391422	SRS2305516	SAMN07268540	412755	marine sediment metagenome	SRA579333
11°N	16S rRNA transcript analysis	SRR5740193	SO248_St8_0-1cm	15.11.2017 23:01	22.06.2017 09:04	45152	26614084	45152	589	13	https://sra-download.ncbi.nlm.nih.gov/traces/sra49/SRR/005605/SRR5740193	SRX2946062	SRP110130	PRJNA391422	391422	SRS2305515	SAMN07268535	412755	marine sediment metagenome	SRA579333
0°S	16S rRNA transcript analysis	SRR5740194	SO248_St6_0-1cm	15.11.2017 23:01	22.06.2017 09:04	51917	30960449	51917	596	16	https://sra-download.ncbi.nlm.nih.gov/traces/sra49/SRR/005605/SRR5740194	SRX2946061	SRP110130	PRJNA391422	391422	SRS2305513	SAMN07268534	412755	marine sediment metagenome	SRA579333
34°N	16S rRNA transcript analysis	SRR5740195	SO248_St12_0-1cm	15.11.2017 23:01	22.06.2017 09:04	43227	24391700	43227	564	12	https://sra-download.ncbi.nlm.nih.gov/traces/sra49/SRR/005605/SRR5740195	SRX2946060	SRP110130	PRJNA391422	391422	SRS2305514	SAMN07268537	412755	marine sediment metagenome	SRA579333
22°N	16S rRNA transcript analysis	SRR5740196	SO248_St10_0-1cm	15.11.2017 23:01	22.06.2017 09:04	55469	32662733	55469	588	16	https://sra-download.ncbi.nlm.nih.gov/traces/sra49/SRR/005605/SRR5740196	SRX2946059	SRP110130	PRJNA391422	391422	SRS2305512	SAMN07268536	412755	marine sediment metagenome	SRA579333
