# Supplementary figures and images for: The Biogeographical Distribution of Benthic Roseobacter Group Members along a Pacific Transect Is Structured by Nutrient Availability within the Sediments and Primary Production in Different Oceanic Provinces
Source: Front Microbiol. 2017 Dec 18;8:2550. doi: 10.3389/fmicb.2017.02550 (PMC5741685; doi:10.3389/fmicb.2017.02550)

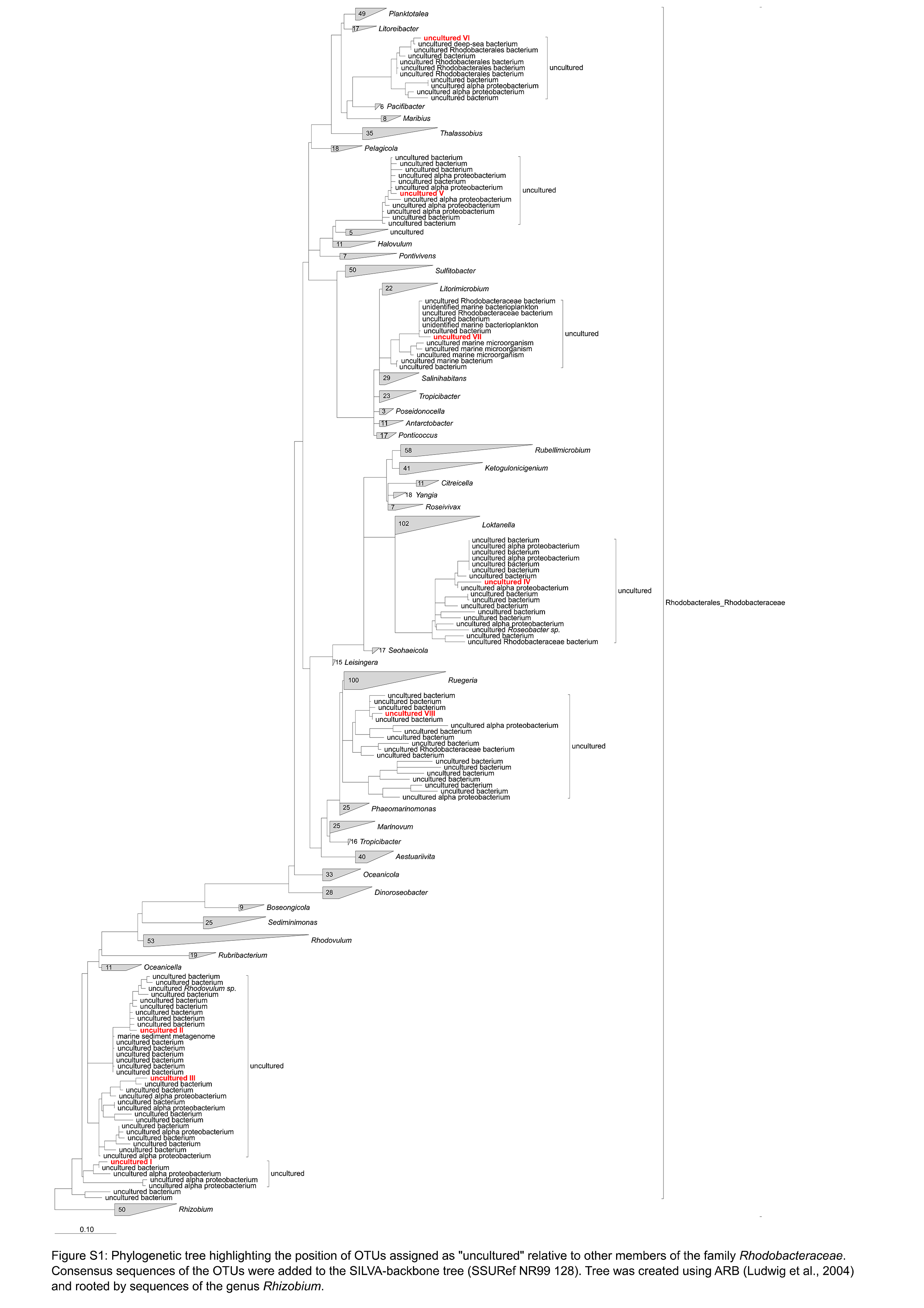

Supplement: Supplementary file 3 [file Image1.TIF]
